# Stress-Induced Grain Refinement in Hard Magnetic Mn_52_Al_45_._7_C_2_._3_ Fabricated Using the Ball-Milling Method

**DOI:** 10.3390/ma15227919

**Published:** 2022-11-09

**Authors:** Seyed Nourallah Attyabi, Seyed Mohammad Ali Radmanesh, Seyyed Ali Seyyed Ebrahimi, Hossein Dehghan, Zahra Lalegani, Bejan Hamawandi

**Affiliations:** 1 Advanced Magnetic Materials Research Center, School of Metallurgy and Materials, College of Engineering, University of Tehran, Tehran 111554563, Iran; 2 Department of Applied Physics, KTH Royal Institute of Technology, SE-106 91 Stockholm, Sweden

**Keywords:** stress-induced, ball milling, MnAl alloys, magnetic properties, ferromagnetic τ-phase, grain refinement

## Abstract

Mn_52_Al_45_._7_C_2_._3_ flakes with different sizes were prepared with two distinct surfactant-assisted ball-milling methods using cylindrical and barrel containers. Different microstructure and magnetic properties were measured based on the sequence of the container shape and different ball-milling times (2, 5, and 10 h). Morphology investigations showed that for powders milled in a barrel container, the amount of τ-phase was more compared to the samples milled in a cylindrical container. Moreover, in the powders milled with barrel containers, considerably higher magnetic properties were obtained in terms of saturation magnetization (M_s_) and remanent magnetization (M_r_) compared to those powders milled with cylindrical containers. Magnetic properties were found to be a function of the ball-milling time. High remanent magnetization and saturation magnetization have been found for powders milled in barrel containers, whereas only mediocre remanent magnetization and saturation magnetization have been measured in the case of milling in cylindrical containers. The highest M_s_ = 52.49 emu g^−1^ and M_r_ = 24.10 emu g^−1^ were obtained for the powders milled in barrel containers for 2 h. The higher magnetic properties taken from the milling in barrel containers is due to the higher shear stress and more uniform strain distribution induced by the barrel configuration, resulting in the stable τ-phase at a reasonably low-strain microstructure.

## 1. Introduction

The τ-phase, introduced as a possible candidate for hybrid cars and electric vehicles in light of cost efficacy, together with superior performance, is becoming more attractive [1,2,3]. A theoretical maximum energy product of 112 kJ m^−3^ (14 MGOe) at room temperature has been reported for rare-earth free MnAl alloys, which could be potentially important materials for permanent magnets [4]. However, the task of reaching this theoretical value proves daunting, as the achieved magnetic properties are not yet comparable with their theoretical magnetic properties. Particle size reduction, as one of the best-known ways for achieving boosted magnetic properties, has been employed and used in fabricating MnAl-and MnAlC-based alloys [2,5,6,7]. The strong impact of this strategy on magnetic properties is believed to come from the surface effects. Up to now, various preparation techniques such as ball milling [6,7], melt spinning [8], magnetron sputtering [9], and mechanical alloying [10] have been used to fabricate MnAlC-based alloys. The lack of control over the particle shape, however, has been detected in almost all these regular techniques. Thus, obtaining pure τ-phase MnAlC alloys with desired characteristics remains a great challenge. On the other hand, more sophisticated procedures such as surfactant-assisted ball milling (SABM) [11,12] and cryogenic milling [3,13] techniques enable the control of the particle morphology and size and have been employed in the fabrication of MnAlC alloys [11,12]. The application of high-energy ball milling as a renowned method with good scalability in making isotropic MnAl powders has been investigated in the literature [14,15,16,17]. This is a common strategy to achieve coercivity, although it is detrimental to magnetization through the application of deforming, as it destabilizes the pseudo-phase τ.

The mechanical milling of the MnAlC alloy effectively reduces the grain size, leading to a significant increase in coercivity from 1.7 kOe to 5 kOe, which is comparable to the highest coercivity reported for MnAlC [18]. The increase in the grain boundary and lattice defects caused by pulverizing also increases the coercivity [19]. The increase in coercivity using the ball-milling method is associated with a significant decrease in residual and saturation magnetizations, which can be due to Mn-Al decomposition during the milling process. The significant decrease in magnetization caused by milling is not only due to the phase transformation [14]. Zijlstra et al. [20] have reported antiferromagnetic coupling at the boundary of lattice defects. They considered the decrease in magnetization to be caused by the accumulation of defects in τ crystals. Shorter Mn-Mn distances lead to antiferromagnetic coupling in the regions adjacent to the defects. Another important reason for the reduction in saturation magnetization is that the milling process leads to an increase in the disorder of the atomic occupation in the MnAl magnetic phase so that more Mn atoms appear in the antiferromagnetic coupling state [21,22].

One of the challenges of MnAl is to achieve high magnetization and a maximum energy product [23] because it is difficult to obtain a high fraction of the τ-phase of MnAl, and also to achieve a microstructure with easy magnetic axis alignment for each grain.

In this paper, we present our recent work on the preparation of MnAlC powders by modified surfactant-assisted high-energy ball-milling (SA-HEBM) techniques in different cylindrical and barrel containers. There is, though, no report on the preparation of hard magnetic MnAlC alloys by a modified ball-milling technique with a barrel container so far. This technique aims to achieve the hard magnetic nanoparticles of MnAlC alloys and to minimize the decomposition of the pseudo-stable critical τ-phase during high-strain milling processes.

## 2. Experimental

### 2.1. Materials and Instrumentation

An MnAlC alloy with the composition of Mn_52_Al_45_._7_C_2_._3_ (at. %) was cast by induction melting in a vacuum of commercial Al, Mn, and C powders. Then, 3 wt.% of Mn powder was added to the composition to compensate for evaporation during the melting process. The resulting τ-MnAl ingot was pulverized and then milled in a planetary ball mill (Fritsch pulverisette).

The actual composition of the casted alloys was analyzed by inductively coupled plasma–optical emission spectroscopy (ICP-OES, 730-ES, Varian, Palo Alto, CA, USA) and carbon content was measured using the LECO CS-244 carbon determinator (ASTM E1019). The structural characterization of the alloys was determined with an X-ray diffractometer (XRD-Rigaku Ultima IV, Cu-Kα). XRD patterns were fitted using the Rietveld method to obtain the lattice parameters, phase percentage, and residual strain for each component. The morphology studies were conducted with a high-resolution scanning electron microscope (FESEM-FEI NOVA NANOSEM 450). The average particle size of MnAlC powders was determined using ImageJ software. Moreover, the particle size distribution was calculated by fitting the data to a Gaussian function in OriginPro software. The magnetic properties of nanoparticles were investigated by a vibrating sample magnetometer (VSM–MagKavCo, 15 kOe).

### 2.2. MnAlC Fabrication Process

**Figure 1:**
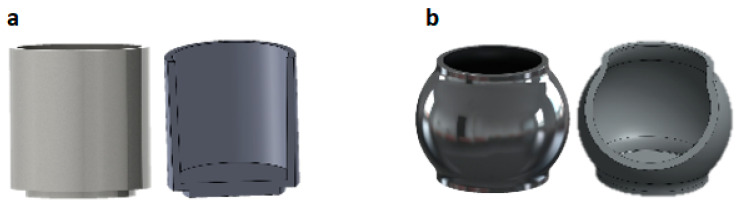
Two types of milling containers: (**a**) cylinder and (**b**) barrel.

Mn_52_Al_45_._7_C_2_._3_ (at. %) alloys were prepared through a vacuum induction melting procedure. The re-melting process was carried out two times under a controlled argon atmosphere to ensure the homogeneity of the casted alloys, as well as to adjust the composition of the obtained parent alloy. It should be noted that the titanium getter system was used to purify the container from residual oxygen. The resultant ingots were then homogenized in a vacuum furnace with *p* = 1 × 10^−4^ Torr at 1000 °C for 5 h along with a subsequent quenching to obtain the critical τ-phase. The final ingots were then crushed and milled in two types of containers: cylindrical and barrel, for different times. The prepared samples are listed in Table 1. Moreover, a schematic of two types of containers is shown in Figure 1. The high-energy ball milling was deployed in cyclohexane as a surfactant with a ball-to-sample weight ratio of 20:1. The resultant magnetic powders were then separated from the surfactant medium via magnetic field separation and subsequently collected. 

## 3. Results and Discussion

### 3.1. Structural Characterization

**Figure 2:**
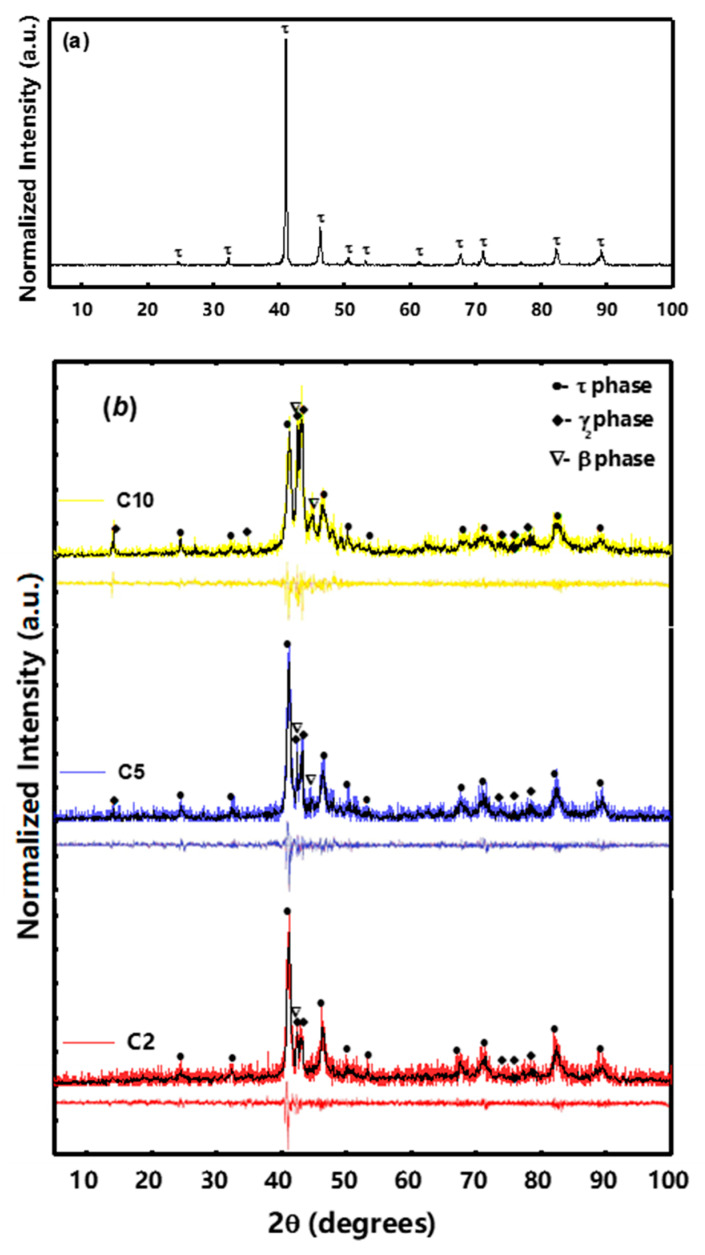

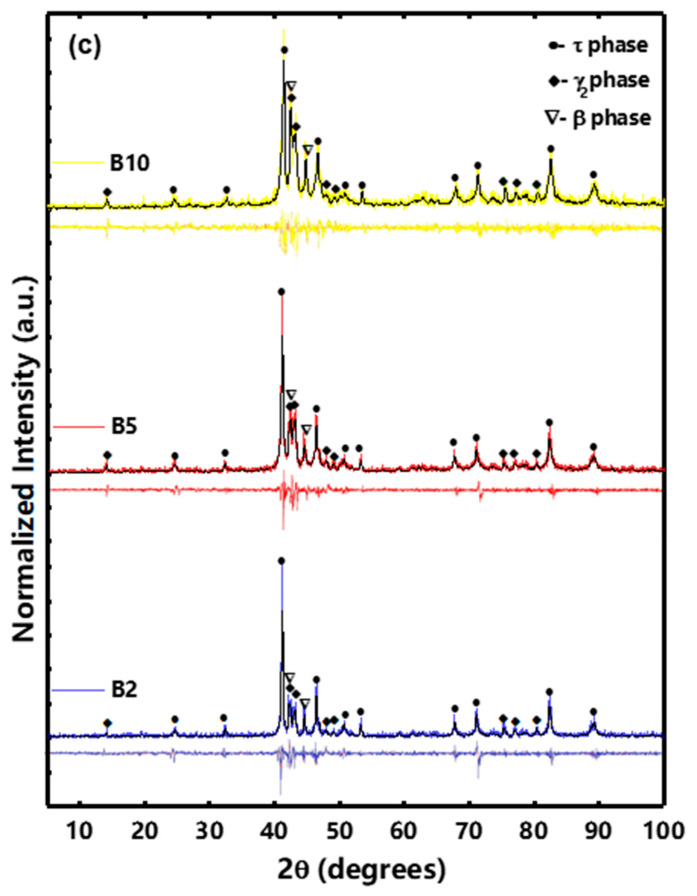
XRD patterns of Mn_52_Al_45_._7_C_2_._3_: (**a**) bulk, (**b**) prepared with a cylindrical container, and (**c**) prepared with a barrel container with different milling times.

Figure 2a–c shows the XRD patterns of bulk and powder samples milled in cylindrical and barrel containers. According to Figure 2a, the bulk MnAlC contains τ-phase without impurity. According to Figure 2b,c, the XRD patterns of milled MnAlC powders in barrel containers exhibit more sustaining τ-phase and less decomposition to the β and γ_2_ phases. The decomposition of the quasi-stable τ-phase in the milling process can be due to the energy transferred from the milling process to the magnetic powders. The variations in lattice parameter, crystallite size, τ-phase percentage, and residual strain were measured using the Rietveld method, as shown in Table 2.

According to Table 2, the axial c/a tetragonality ratio for the samples obtained with barrel containers is higher than that for the samples obtained with cylindrical containers. During the initial process of milling in the barrel container, there is no significant lattice strain or distortion. With the increase in milling time, a high strain is created in the powders and lattice changes are increased. This reduces the c/a ratio as well as the residual strain, highlighting the correlating effect between the lattice parameter and the residual strain in the barrel container. In contrast, for cylindrical containers, the residual strain rapidly increases in longer milling times, which possibly peaks and eventually decreases. The stronger impact forces in the cylindrical container generate a smaller crystalline size with higher levels of defect and disorder in the structure. The relative long-range order parameter for τ-phase, S, was calculated separately when the barrel and cylindrical containers were used, respectively. The procedure results in the comparison of the relative peak intensity of the superlattice (h + k + l = odd) and fundamental structure (h + k + l = even), as shown below [24,25,26]:

(1) $$S=\sqrt{\frac{{\left(I_{s}/I_{f}\right)}_{dis.}}{{\left(I_{s}/I_{f}\right)}_{ord.}}}$$

where *I_s_* and *I_f_* are the peak intensity of the superlattice and fundamental structure, respectively.

According to Table 2, the long-range order is much more prominent in barrel containers than that in cylindrical containers. Both containers give a smaller S parameter for longer milling times, but the difference is more severe in cylindrical containers.

### 3.2. Morphology Investigation

**Figure 3:**
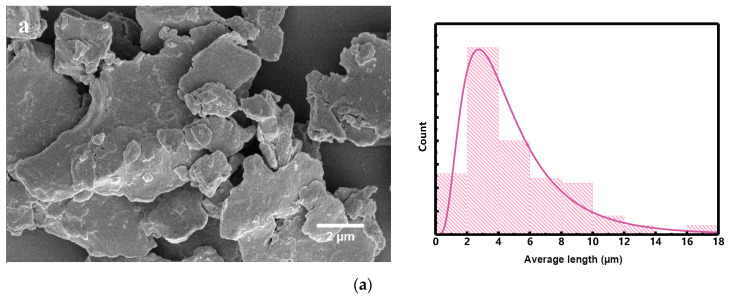

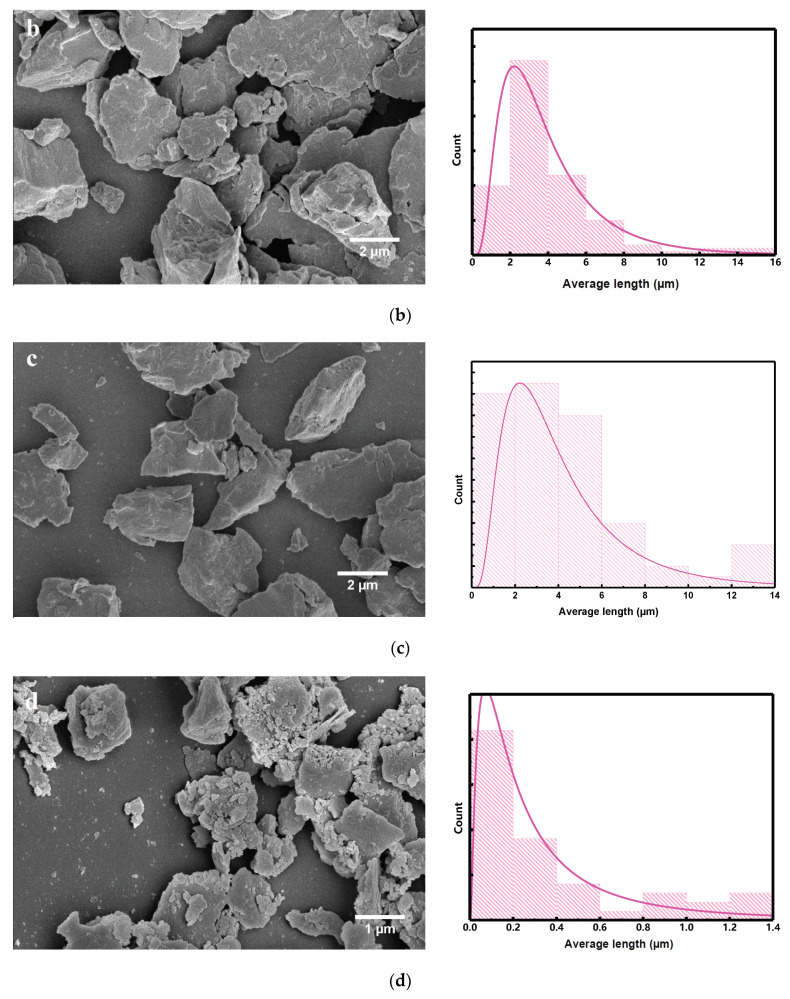

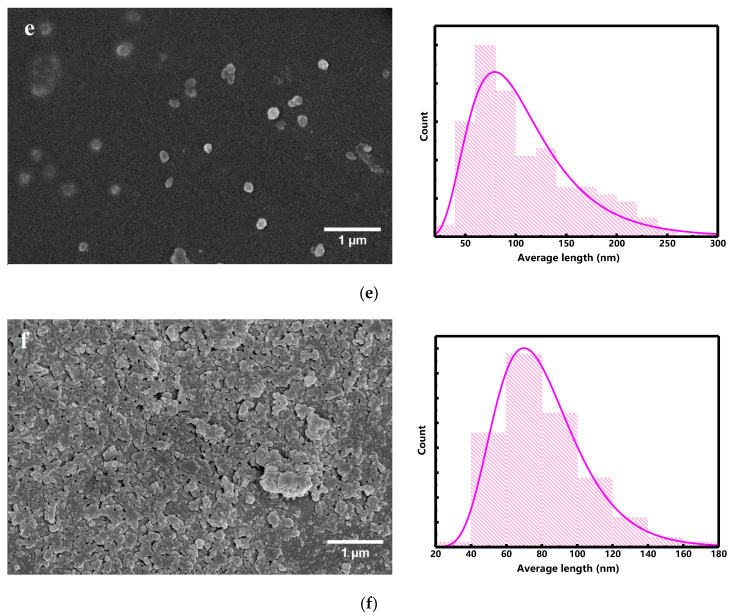
FESEM images and histograms of the particle size distribution of Mn_52_Al_45_._7_C_2_._3_ milled at different times and containers: (**a**) 2 h cylindrical, (**b**) 5 h cylindrical, (**c**) 10 h cylindrical, (**d**) 2 h barrel, (**e**) 5 h barrel, and (**f**) 10 h barrel.

Figure 3a–f displays the FESEM images and particle size distribution histogram of the Mn_52_Al_45_._7_C_2_._3_ powders milled in the cylindrical and barrel containers. For the cylindrical container, the milled powders are several micron particles in size and the shape of flakes with cracks in some regions, which shows the predominance of impact forces in this process. In the initial steps of the milling, the repeating impact forces transferred to the powders cause compressive strain, resulting in the flake-like shapes of the powders. In longer milling times, the grain size distribution narrows while the powder shape is unchanged (Figure 3c).

FESEM images and histograms of the milled powders in barrel containers show smaller mean grain sizes than powders milled in cylindrical containers. As can be seen in Figure 3d (B2 sample) the powders’ surfaces are step-like and porous. In addition, pieces of nanoparticles have aggregated on the microparticles and in the background, which shows the predominance of shearing forces compared to impact forces. In longer milling times, the shear forces break down the initial particles into Mn-Al-C nanoparticles. For very long milling times (10 h), the concurrent shear and impact forces render bonding in nanoparticles, which causes the necking of the particles, as shown in Figure 3f (B10 sample).

**Figure 4:**
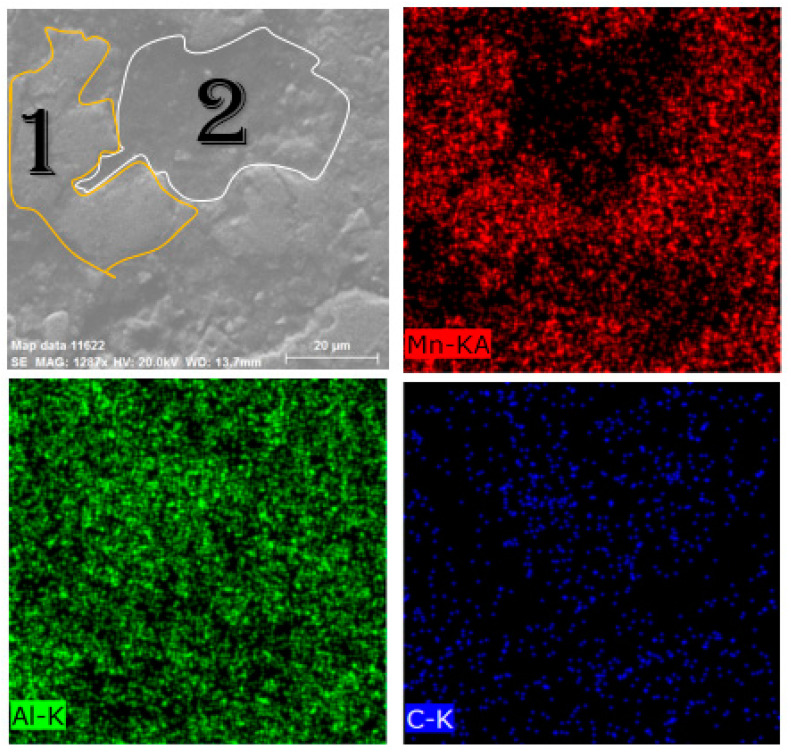
Morphology and mapping images of milled sample in the cylindrical container.

**Figure 5:**
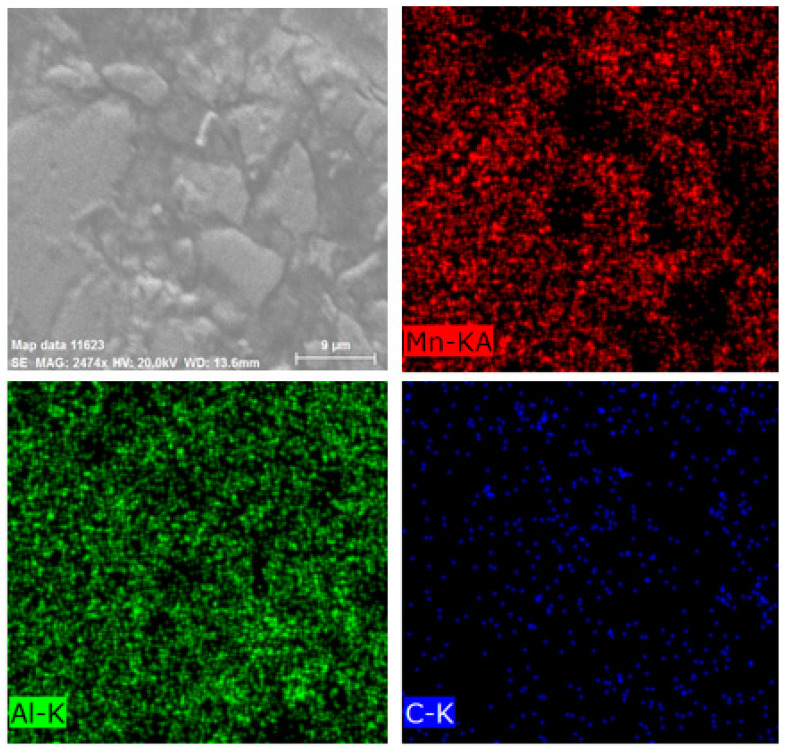
Morphology and mapping images of milled sample in the barrel container.

To observe the τ-phase and confirm the XRD results, EDS analysis was performed on the milled samples using cylindrical and barrel containers. For this purpose, the samples were first pressed and mounted, and then they were etched in a solution of hydrofluoric acid (1%), nitric acid (3%), hydrochloric acid (6%), and water (90%). Figure 4 shows the morphology and mapping images of the milled sample in the cylindrical container. According to the map image of Mn (Figure 4), the Mn in region 1 is much higher than in region 2. On the other hand, the amount of Al in region 2 is higher than in region 1. EDS analyses of these two regions were performed, as shown in Figures S1 and S2. According to Figure S1, in region 1, the weight percentage of Mn (~62 wt.%) is much higher than that of Al (~26 wt.%). As well, it can be seen in Figure S2 that in region 2 the weight percentage of Al (~45 wt.%) is much higher than Mn (~16 wt.%). Therefore, it can be concluded that region 1 represents the τ-phase and region 2 represents the γ_2_ phase. Figure 5 shows the morphology and mapping images of the milled sample in the barrel container. As can be seen in Figure 5, more regions are composed of τ-phase and compared to the sample milled with a cylindrical container, it has less γ_2_ phase.

### 3.3. Magnetic Property Investigation

**Figure 6:**
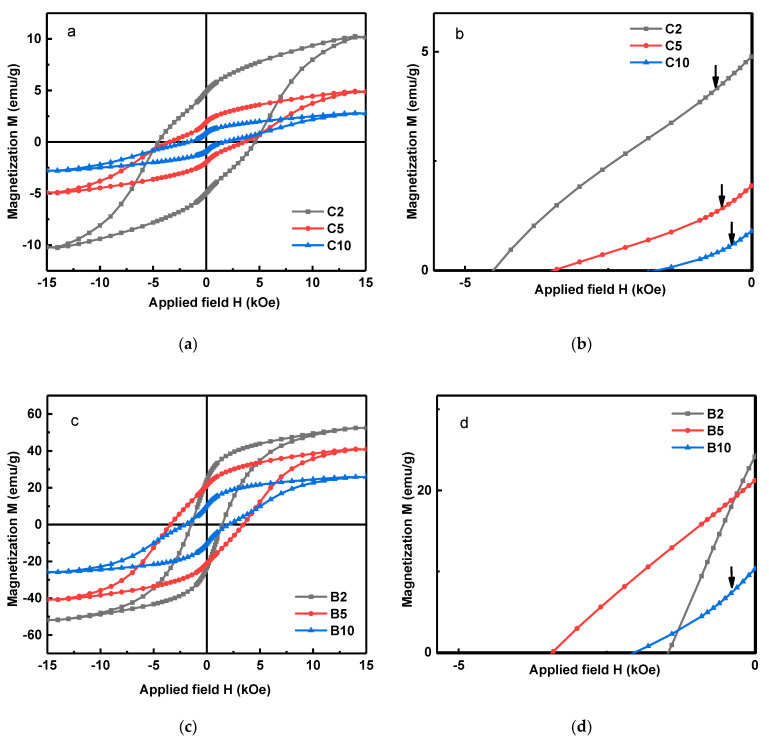
M-H hysteresis loops for Mn_52_Al_45_._7_C_2_._3_ samples prepared by (**a**,**b**) cylinder container and (**c**,**d**) barrel container at different milling times (t = 2, 5, and 10 h); the arrows show the kinks caused by defects.

Figure 6 shows the hysteresis loops for the milled powders in the barrel and cylindrical containers at different milling times. The magnetic properties of Mn_52_Al_45_._7_C_2_._3_ milled in different conditions provide more pieces of evidence for the procedural influence on the microstructure and stress-treated particles.

In all cases, the barrel container led to much higher magnetic properties, such as M_r_ and M_s,_ shown in Table 3. This could be attributed to the seemingly larger fraction of the ordered phase τ resulting from the barrel container. Now, we focus on the microstructural influence on magnetic properties. In the demagnetizing curves of powders in the cylindrical container and also in B10, the emerged kink (Figure 6d) is caused by the elevated density of defects such as lattice distortion, stacking faults, and dislocations due to milling-driven stresses. A similar kink has been reported in the literature to be caused by the strains induced during the milling process [5,6,11].

**Figure 7:**
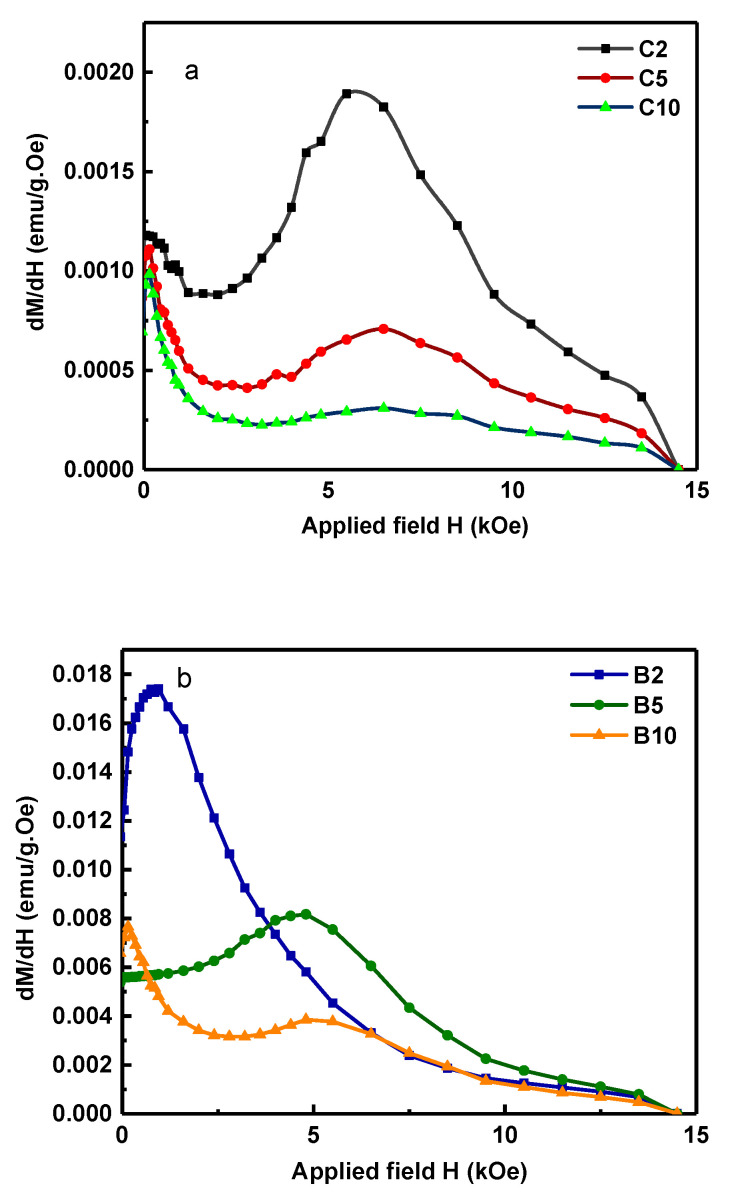
First-order derivative dM/dH of the hysteresis loops for Mn_52_Al_45_._7_C_2_._3_ samples prepared with the (**a**) cylinder container and (**b**) barrel container at different milling times (t = 2, 5, and 10 h).

The forces introduced to powders in the cylindrical containers increase the density of defects in the samples and the kink appears in the demagnetization curve. On the other hand, the defects, acting as pinning centers, increase the coercivity of powders milled in the cylindrical container [27,28,29,30,31,32]. According to Table 3, a noteworthy coercivity of 4.53 kOe has been obtained for C2. Figure 7 shows the first-order derivative of the hysteresis loops (dM/dH) for the B and C samples.

According to Figure 7b, the demagnetizing curves of samples B2 and B5 are smooth curves that show the reduction in defect density in these samples during the milling process. For the formation of MnAlC flakes, micron-sized powders are formed with the increase in the internal strain of the particles due to milling. Then, these particles are broken apart due to the sliding of basal planes and form sub-micron flake particles. Defects made by the milling process cause stacking fault defects on flake particles and lead to increased coercivity. By reducing the size of the particles to nano in the barrel container corresponding to sample B5 (Figure 3e), due to the lack of a magnetic wall in the single-domain particles, the mechanism of demagnetization is the rotation of domains; by applying an external field, the direction of magnetization moves from the easy axis to the hard axis. To move the magnetization vector during the rotation process from the hard direction to the new stable direction, it is necessary to apply a stronger field; therefore, this leads to an increase in coercivity, with a coercivity of 3.42 kOe obtained for sample B5.

The coercivity of 3.42 kOe for B5 indicates the magnetic domain’s rotation as the dominant demagnetizing mechanism, concluded from the particle’s size. In addition, the defect density reduction inhibits the anti-ferromagnetic coupling between neighboring Mn atoms, increasing the remanent as well as saturation magnetization. Moreover, the demagnetizing trends can be evaluated using the derivative hysteresis curves for different milling cases in the barrel and cylindrical containers (Figure 7). The derivative hysteresis curves reveal the domain’s switching field (H_sw_). Typically, the ideal single-step magnetization reversal is achieved when a single peak is observed, and the goal is to produce a material with a high switching field and a narrower switching field distribution (SFD).

According to Figure 7b, the derivative hysteresis curves for B2 and B5 indicate merely single peaks, showing the exchange coupling of particles. On the other hand, in derivate hysteresis loops for samples milled in cylindrical containers as well as in B10, two peaks are observed. This can be attributed to the higher density of defects, and hence the lack of exchange coupling. It is worth mentioning, except for B2, all derivative hysteresis curves look symmetric. In contrast, the derivative hysteresis loop for B2 is rather asymmetric and its peak does not correspond to the coercivity value of the M(H) diagram. One reason for this trend could be the inhomogeneous defect density. Having less transferred energy to particles in barrel containers and the existence of micrometer particles for B2, revealed from the XRD and FESEM, causes high concentrations of defects to develop near the surface, leaving the particle cores almost defect-free. This, in turn, generates inhomogeneous properties. Near-surface regions with high concentrations of defects display larger switching fields due to the domain wall pinning. However, a prominent fraction of the domain walls rotates at a rather small H_sw_ due to the low defect density. 

A very high level of transferred energy during milling in cylindrical containers renders a sort of homogeneous concentration of defects throughout the particles, resulting in a more symmetric derivative curve. For B5 and B10 samples, the drastic reduction in particle size has resulted in a uniform defect density, and a more symmetrical hysteresis derivative curve is observed that also matches the coercivity field.

SFD is calculated according to $FD=\Delta H/H_{c}$, where ΔH is the width at half the maximum peak of the *dM/dH* curve, and *dM/dH* is obtained by differentiating the hysteresis loop. SFD can be used to determine the degree of squareness of the hysteresis curve, such as $M_{r}/M_{s}$. A smaller SFD coming with a narrower half-peak *dM/dH* is equivalent to better curve squareness, which describes the case of B2 well. 

The rather narrow SFD for the derivative hysteresis curve in the case of B2 originates from a narrower particle size distribution in the barrel container, making the particles’ coercivities comparable. This demonstrates that for B2 the magnetic particles have transformed from multi-domains to single and pseudo-single domains. 

One should note that the ball-milling process generates impact, shear, and frictional forces with several acting factors on them [33,34]. The type of milling (low energy, high energy), the milling time, and the temperature of heat treatment after milling are parameters that affect the magnetic properties. It should be noted that, in many reports, heat treatment has been applied after milling to reduce the strains of the crystal lattice, which increases the magnetization of the material [12,15,28,31,35]. In the present work, the effect of different induced stresses in the milling has been investigated, and heat treatment has not been applied. Table 4 shows a comparison between the magnetic properties of MnAl alloys obtained via milling with and without heat treatment.

According to the theory of plasticity, the principal stresses applied to the powders in the barrel container lead to higher shear stress. This can be ascribed to the higher hydrostatic stress and the more even stress distribution in the body of the barrel container, implying greater shear stress during the milling process. This can be precisely explained through the two-dimensional stress analysis of Mohr’s circle as [36]:

(2) $$R={\left[{\left(\frac{\sigma_{x}-\sigma_{y}}{2}\right)}^{2}+\tau_{xy}^{2}\right]}^{\frac{1}{2}}$$

where *σ_x_* and *σ_y_* are principal tensions and *τ_xy_* is shear stress. Moreover, in the case of three dimensions, the shear stress can be derived as:

(3) $$\tau_{1}:\tau_{2}:\tau_{3}=\frac{1}{2}\left|\sigma_{1}-\sigma_{2}\right|:\;\frac{1}{2}\left|\sigma_{2}-\sigma_{3}\right|:\;\frac{1}{2}\left|\sigma_{3}-\sigma_{1}\right|$$

To hold the assumption of the constant volume during plastic deformation $=d\varepsilon_{x}+d\varepsilon_{y}+d\varepsilon_{z}=0$, the following representative equations are used for the real strains [37]:

(4) $$d\varepsilon_{1}:d\varepsilon_{2}:d\varepsilon_{3}=\left[\sigma_{1}-\frac{1}{2}\left(\sigma_{2}+\sigma_{3}\right)\right]:\left[\sigma_{2}-\frac{1}{2}\left(\sigma_{3}+\sigma_{1}\right)\right]:\left[\sigma_{3}-\frac{1}{2}\left(\sigma_{1}+\sigma_{2}\right)\right]$$

which indicates the relative actual strains in principal directions due to the plane symmetry. This subsequently induces even strains in all principal directions, to further control the shape and morphology of the particles more effectively. This can be confirmed by going through the FESEM images of the milled powders in the barrel container (Figure 3d–f). 

Therefore, the decomposition of the critical τ-phase has been moderated in the barrel container, producing very high magnetic characteristics. Additionally, the variation in coercivity with preserving the ferromagnetic τ-phase has been improved in B5. The B5 milled powders in this case benefit from the critical fine-grained τ-phase governing the demagnetizing behavior. However, in the cylindrical container, the decomposition of the τ-phase has been intensified by the high level of strains applied to the powders. 

## 4. Conclusions

In this work, the mechanical milling was performed in two different containers to investigate the effectual approach for minimizing the τ-phase decomposing during the high-strain-contained milling. It turned out that medium strains implicate less τ-phase, decomposing with a high degree of chemical ordering. These features would be declined in ultra-high applied strains, which means the high levels of micro-strain reduce the activation energy of the immediate neighboring Mn-Mn atoms. The diminished XRD intensity and peak width along with the deteriorated order parameter of the τ-phase against the milling time support the idea of sensitivity in the pseudo-stable τ-phase to the high levels of strain. Hence, less strain and stronger shear stresses may be employed to improve the magnetic properties. Based on the c/a parameter obtained from the XRD analysis, it was expected that due to the higher tetragonality (c/a) in the barrel container samples, a higher coercivity would be obtained in these samples, but due to the great density of defects as pinning centers in the cylindrical containers, the coercivity for these samples was up to 4.53 kOe. However, a high density of defects reduces the lattice parameter, promotes the anti-ferromagnetic coupling between Mn atoms in the grains rich with Mn, and reduces the magnetization. 

The highest M_s_ = 52.49 emu g^−1^ and M_r_ = 24.10 emu g^−1^ were reported for B2, having the best chemical ordering and the largest τ-phase fraction. Nevertheless, B5 had good intergranular magnetic exchange coupling among the τ-phase grains, which illustrates the decent magnetic features of M_s_ = 40.93 emu g^−1^, M_r_ = 21.31 emu g^−1^, H_c_ = 3.42 kOe and M_r_ / M_s_ = 0.52.

## Figures and Tables

**Table 1:** Alloy samples with different milling container types and times.

Milling Container Type	Milling Time (h)	Sample Name
Cylinder	2	C2
	5	C5
	10	C10
Barrel	2	B2
	5	B5
	10	B10

**Table 2:** Summary of the refined XRD parameters for the Mn_52_Al_45_._7_C_2_._3_ samples after milling in cylindrical and barrel containers for 2, 5, and 10 h.

Sample	Phase	c/a	Phase Content (%)	Strain (%)	Size (nm)	Goodness of Fit (GOF)	S
C2	τ	1.298	83.2	0.1	51.10	1.98	0.801
	β, γ_2_		16.8				
C5	τ	1.296	76.1	0.4	39.62	1.76	0.795
	β, γ_2_		23.9				
C10	τ	1.295	47.7	0.89	31.92	1.96	0.754
	β, γ_2_		52.3				
B2	τ	1.299	92.4	0.1	76.34	1.06	0.907
	β, γ_2_		7.6				
B5	τ	1.301	86.2	0.3	57.92	1.03	0.872
	β, γ_2_		13.8				
B10	τ	1.300	75.3	0.2	37.01	1.10	0.816
	β, γ_2_		24.7				

**Table 3:** Magnetic properties for the Mn_52_Al_45_._7_C_2_._3_ samples after milling in cylindrical and barrel containers for 2, 5, and 10 h.

Sample	M_s_ (emu g^−1^)	M_r_ (emu g^−1^)	M_r_/M_s_	H_c_ (kOe)	H_SW_ (kOe)
C2	10.29	4.87	0.47	4.53	5.78
C5	4.98	1.92	0.38	3.51	6.5
C10	2.8	0.9	0.32	1.64	6.48
B2	52.49	24.10	0.45	1.47	0.94
B5	40.93	21.31	0.52	3.42	4.71
B10	25.89	10.46	0.40	2.01	4.97

**Table 4:** Comparison of magnetic properties of MnAl alloys with different milling methods.

Alloy	Milling Type	Particle Shape	M_r_ (emu g^−1^)	H_c_ (kOe)	Ref.
Mn_54_Al_46_	SA-HEBM (30 s, 900 rpm, 340 °C)	-	5	4.2	[35]
Mn_54_Al_46_	SA-HEBM (3 min, 900 rpm, 350 °C)	Flake	10	4.5	[31]
Mn_54_Al_46_	SA-HEBM (30–270 s, 900 rpm, 340 °C)	Flake	5–7	4–4.5	[28]
Mn_54_Al_46_	SABM	Flake	-	3	[12]
Mn_54_Al_43_C_3_	SPEX 8000	Flake	5≤	4.6	[15]
Mn_52_Al_45_._7_C_2_._3_	SA-HEBM (cylindrical container, without annealing)	Flake	4.87	4.53	This work
Mn_52_Al_45_._7_C_2_._3_	SA-HEBM (barrel container, without annealing)	Nanoparticle	21.31	3.42	This work

## Data Availability

The data presented in this study are available on request from the corresponding author of first author.

## Supplementary Materials

The following supporting information can be downloaded at: https://www.mdpi.com/article/10.3390/ma15227919/s1. Figure S1. EDS analysis and weight percentage of Mn and Al for the region 1 of Figure 4. Figure S2. EDS analysis and weight percentage of Mn and Al for the region 2 of Figure 4.
