# Supplementary material for: Stress-Induced Grain Refinement in Hard Magnetic Mn52Al45.7C2.3 Fabricated Using the Ball-Milling Method
Source: Materials (Basel). 2022 Nov 9;15(22):7919. doi: 10.3390/ma15227919 (PMC9694233; doi:10.3390/ma15227919)
Supplement: Supplementary file 1 [file materials-15-07919-s001.zip › materials-1983905-supplementary.pdf]

Supplementary material for

Stress-Induced Grain Refinement in Hard Magnetic $Mn_{52}Al_{45.7}C_{2.3}$ Fabricated Using the Ball-Milling Method

Seyed Nourallah Attyabi [1], Seyed Mohammad Ali Radmanesh [1], Seyyed Ali Seyyed Ebrahimi [1,*], Hossein Dehghan [1], Zahra Lalegani [1] and Bejan Hamawandi [2,*]

[1] Advanced Magnetic Materials Research Center, School of Metallurgy and Materials, College of Engineering, University of Tehran, Tehran 111554563, Iran

[2] Department of Applied Physics, KTH Royal Institute of Technology, SE-106 91 Stockholm, Sweden

* Correspondence: saseyyed@ut.ac.ir (S.A.S.E.); bejan@kth.se (B.H.)

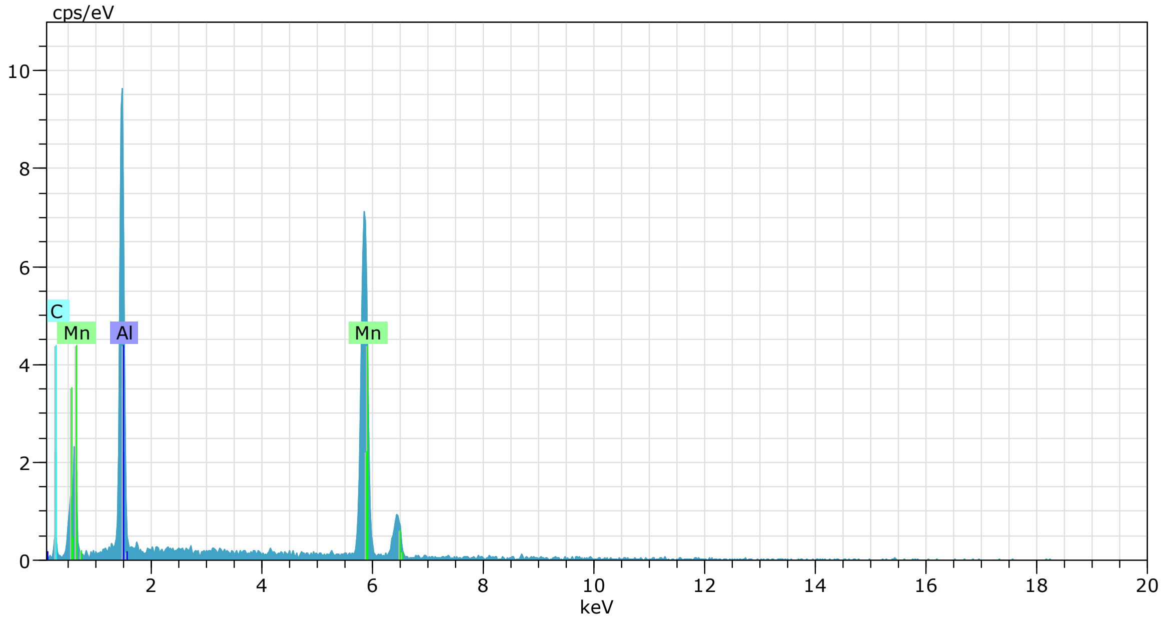

El	AN	Series	unn. C [wt.%]	norm. C [wt.%]	Atom. C [at.%]	Error (1 Sigma) [wt.%]
Mn	25	K-series	48.93	62.43	37.01	1.56
Al	13	K-series	20.25	25.84	31.18	1.11
C	6	K-series	9.20	11.73	31.81	4.15
Total:			78.38	100.00	100.00	

Figure S1. EDS analysis and weight percentage of Mn and Al for the region 1 of Figure 4.

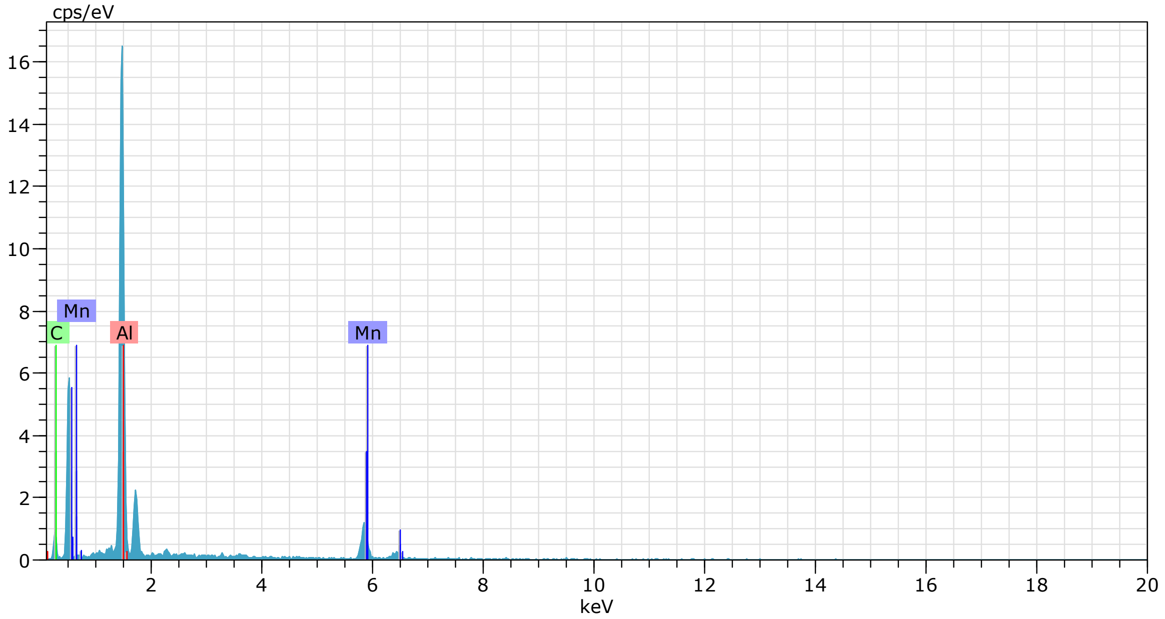

El	AN	Series	unn. C [wt.%]	norm. C [wt.%]	Atom. C [at.%]	Error (1 Sigma) [wt.%]
Al	13	K-series	29.29	45.46	32.67	1.52
C	6	K-series	24.56	38.11	61.53	8.43
Mn	25	K-series	10.59	16.43	5.80	0.52
		Total:	64.43	100.00	100.00	

Figure S2. EDS analysis and weight percentage of Mn and Al for the region 2 of Figure 4.
